# The Clinical and Metabolic Profiles in Menstrual Changes Among Reproductive‐Aged Women Post‐COVID‐19

**DOI:** 10.1002/mco2.70240

**Published:** 2025-06-11

**Authors:** Wei Wang, Manfei Si, Xinyu Qi, Hongxia Hu, Xiaole Sun, Juyan Liang, Jianghua Zhou, Xianmin Bi, Wei Zhao, Yuanyuan Wang, Liying Yan, Rong Li, Wei Chen, Jie Qiao

**Affiliations:** ^1^ Department of Obstetrics and Gynecology Peking University Third Hospital Beijing China; ^2^ National Clinical Research Center for Obstetrics and Gynecology (Peking University Third Hospital) Beijing China; ^3^ Center for Reproductive Medicine, Department of Obstetrics and Gynecology Peking University Third Hospital Beijing China; ^4^ State Key Laboratory of Female Fertility Promotion, Department of Obstetrics and Gynecology Peking University Third Hospital Beijing China; ^5^ Physical Examination Center Peking University Third Hospital Beijing China; ^6^ Beijing Advanced Innovation Center for Genomics Beijing China; ^7^ Peking‐Tsinghua Center for Life Sciences Peking University Beijing China

**Keywords:** COVID‐19, hormone, menstrual change, metabolomics, recovery duration

## Abstract

Menstruation is a key indicator of female reproductive health, yet clinical features and underlying mechanisms associated with menstrual changes following the coronavirus disease 2019 (COVID‐19) infection remain unclear. Here, we recruited 253 participants through questionnaires, and 73 individuals underwent metabolomic analysis of blood serum. Over 60% reported menstrual changes, primarily experiencing longer cycle and lighter bleeding, which were significantly associated with age, general medical conditions, perceived stress, anxiety scores, and depression scores, as well as COVID‐19 symptoms including fatigue and headache. General medical conditions were the sole independent risk factor for any menstrual changes. Metabolomic analysis highlighted disturbances in steroid hormone biosynthesis. We identified 52 significantly differential metabolites between groups with and without any menstrual changes (AnyC vs. NoC), with high discrimination achieved by combining phenylglyoxylic acid, PC O‐40, traumatic acid, and estrone sulfate. Furthermore, several significantly upregulated metabolites were closely correlated with estradiol (E_2_) levels, including estrone sulfate, which was also positively correlated with T levels. Specifically, T levels decreased with recovery duration in the AnyC group (*p* = 0.0015). Collectively, our findings uncovered key clinical factors and metabolic disruptions in menstrual changes, underscoring potential adverse long‐term effects of COVID‐19 on women's health.

## Graphical abstract

This study recruited 253 Chinese women of reproductive age and post‐COVID‐19 infection. Serious factors, including age and perceived stress, contribute to menstrual changes while general medical condition was the only independent risk factor. Notable metabolic change existed in group with any menstruation changes, with special sex hormone: T decreased with recovery duration.

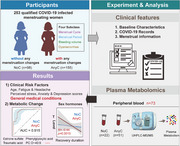

## 1 Background

The coronavirus disease 2019 (COVID‐19), caused by severe acute respiratory syndrome coronavirus 2 (SARS‐CoV‐2), broke out and rapidly spread globally since December 2019 [1]. Over the past few years, it has generated significant concern and panic globally due to its high contagiousness [2]. Three years later, on December 7, 2022, China discontinued its national dynamic zero‐COVID strategy and relaxed its prevention and control measures for COVID‐19 [3, 4]. As a result, almost the entire country was infected. The clinical manifestations of COVID‐19 primarily include fever, malaise, dry cough, however, some patients may experience nasal congestion, runny nose, diarrhea, and other symptoms [5]. Severe cases often develop respiratory distress after 1 week, with rapid progression to acute respiratory distress syndrome, septic shock, difficult‐to‐correct metabolic acidosis, and hemorrhagic coagulopathy [6]. COVID‐19 has been reported as a systemic disorder that can affect the respiratory, cardiac, ocular, urologic, digestive, and neurologic systems [7].

Many concerns have been raised regarding the effects of COVID‐19 on reproductive health [8], particularly among women of reproductive age [9]. Regular menstruation serves as a crucial indicator of female physiological health and reproductive maturity [10, 11]. There are numerous reasons for menstrual changes, including gynecological disorders (e.g., polycystic ovary syndrome, premature ovarian insufficiency, endometrial polyps, etc.), physiological aging, excessive weight loss or exercise, psychological stress, unhealthy habits, including smoking and drinking, and vaccination [12]. Following the COVID‐19 pandemic, numerous studies from various countries have reported alterations in menstruation [13, 14, 15, 16]. Most surveys were cross‐sectional studies that identified an increase in menstrual changes through online questionnaires [17, 18, 19]. A systematic review summarized 16 articles indicating that women experiencing COVID‐19 infection or pandemic‐associated stress and anxiety were more likely to encounter menstrual disorders or irregularities [13], in line with the findings of Li et al. [20]. According to a study by Nguyen et al., menstrual changes manifested as decreased cycle length and increased menstrual duration, with a higher incidence of anovulatory cycles and abnormal cycle lengths among women [21]. Conversely, a review by Lebar et al. indicated that menstrual changes primarily involved decreased menstrual volume and prolonged cycles following COVID‐19 infection [22]. Muharam et al. reported an increase in patients experiencing changes in cycle length, menstrual irregularities, and new cases of heavy menstrual bleeding subsequent to COVID‐19 infection [23]. Overall, most studies have reported irregular menstruation and prolonged menstrual cycles [24, 25, 26, 27]. In contrast, some studies reported a reduction in menstrual problems during the pandemic [28]. Following the coronavirus outbreak after the termination of the “dynamic zero‐COVID policy,” there appears to be an increase in the proportion of women visiting gynecology clinics due to abnormal menstruation, raising concerns among the Chinese population regarding the association between COVID‐19 and menstrual changes. The key factors that triggered abnormal menstruation following COVID‐19 infection require comprehensive analysis.

Moreover, while earlier investigations have delineated various impacts of COVID‐19 infection on female menstruation, research on the molecular mechanisms induced by COVID‐19 leading to menstrual irregularity is rarely reported. SARS‐CoV‐2 infection may influence the menstrual cycle through both psychological and biological pathways, either via direct interactions with reproductive tissues or indirectly through stress and inflammation [29, 30]. Previous studies have reported undetectable viral RNA in endometrial tissue samples from SARS‐CoV‐2‐positive women, and no acute or chronic inflammatory lesions of the endometrium were identified histologically through hematoxylin–eosin staining [31, 32]. However, Henarejos‐Castillo et al. suggested that COVID‐19‐related gene expression changes affect endometrial receptivity and key processes essential for fertility [33]. Metabolomics has been widely employed to explore the underlying biological processes and mechanisms related to menstruation or reproduction [34, 35, 36]. Numerous metabolomic studies have indicated metabolic abnormalities in individuals recovering from COVID‐19 or those with Long COVID syndrome [37, 38, 39]. Notably, Lomova et al. identified significant alterations in the lipid profiles of follicular fluid in women with a history of SARS‐CoV‐2 infection, which may predict the risk of miscarriage in subsequent pregnancies [40]. Therefore, these metabolic changes likely represent an important molecular mechanism mediating COVID‐19‐related menstrual alterations.

Additionally, long‐term COVID symptoms have been associated with hormonal imbalances [41]. Observational studies indicate that ovarian function is suppressed during the acute phase of SARS‐CoV‐2 infection, which seems to disrupt ovarian hormone regulation, ultimately leading to menstrual changes [42]. Thus, in addition to menstrual changes, markers of ovarian reserve, such as sex hormones, including anti‐Müllerian hormone (AMH), follicle‐stimulating hormone (FSH), luteinizing hormone (LH), E_2_, progesterone (P), testosterone (T), prolactin (PRL), and androstenedione (AND) represent another important component to be evaluated [43, 44]. Current studies indicated short‐term fluctuations in key ovarian reserve markers, including a decrease in AMH and LH and an increase in FSH and PRL [44, 45]. Although these markers appear to normalize during the recovery period, suggesting that ovarian dysfunction is not a permanent change, the potential for long‐term adverse effects should not be overlooked [44]. Furthermore, the relationship between variations in ovarian marker levels during recovery and abnormalities in menstrual cycle‐related indicators warrants further investigation.

Since nearly all women in mainland China experienced their first infection immediately following the termination policy of “dynamic zero‐COVID policy” [46], this provides an exceptionally rare and valuable opportunity, to assess the long‐term impacts of COVID‐19 on menstruation and ovarian function without the confounding effects of prior infection, which are often unavoidable in other cohorts. The present study aims to investigate the effects of COVID‐19 infection on menstrual status and reproductive endocrine disorders, and to elucidate the underlying molecular mechanisms through metabolomics.

## 2 Results

### 2.1 The Global Features of Recruited Population

**FIGURE 1:**
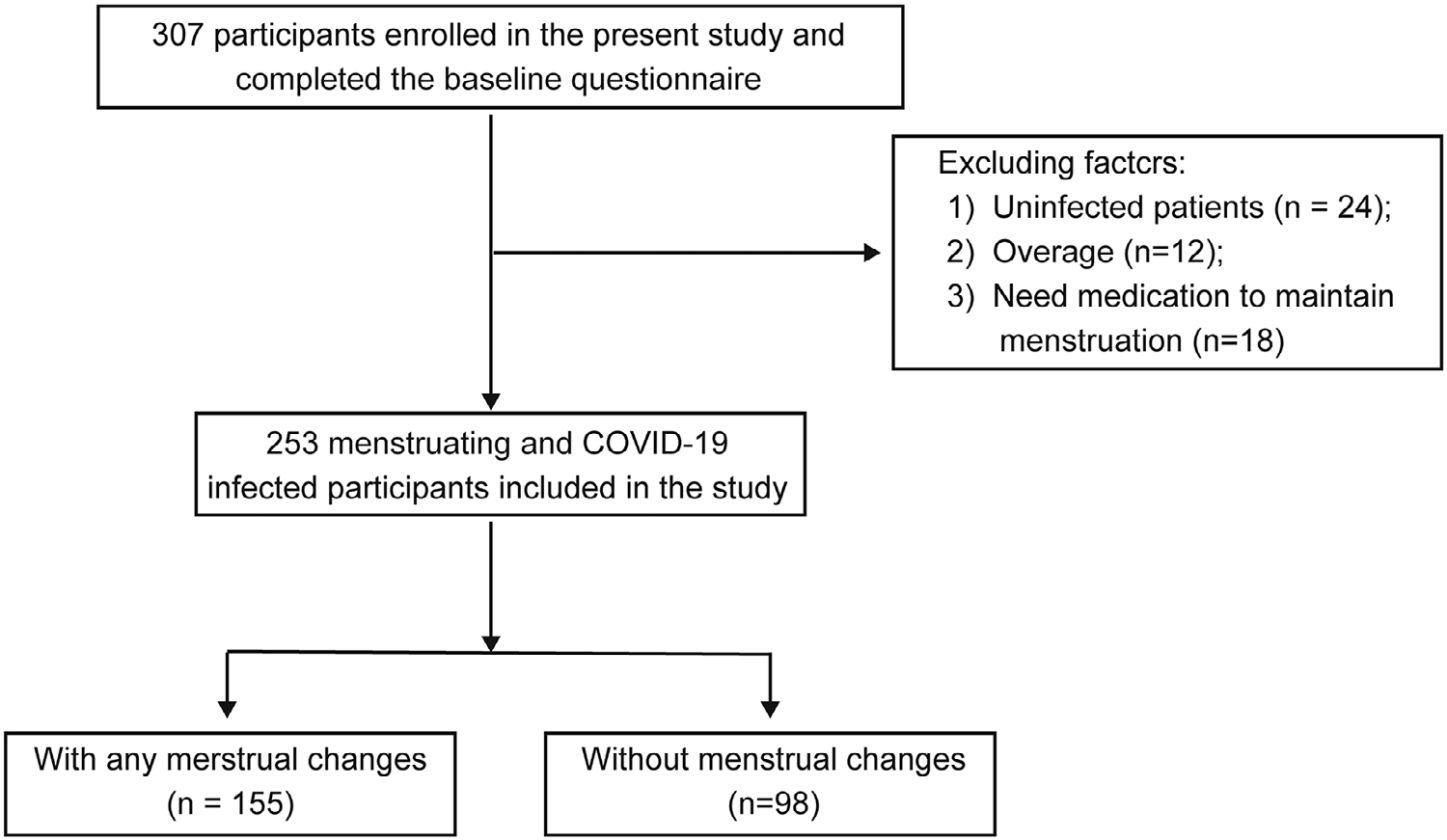
Flowchart of the study population. This flowchart illustrates the participant selection process and distribution in a study on menstrual changes among individuals infected with COVID‐19. Initially, 307 participants were enrolled and completed the baseline questionnaire. After excluding 24 uninfected participants, 12 overweight individuals, and 18 individuals who needed medication to maintain menstruation, 253 menstruating and COVID‐19‐infected participants were included in the study. Among these, 155 participants experienced any menstrual changes, while 98 did not.

In total, 307 women participated in this survey. Among these women, 7.82% (*n* = 24) were not infected. A total of 30 women (9.77%) were excluded from the study due to exceeding the age criteria (*n* = 12) or requiring medication to maintain menstruation (*n* = 18). Finally, a total of 253 menstruating women diagnosed with COVID‐19 were included in our cohort (Figure 1).

### 2.2 Clinical Characteristics of Women With COVID‐19 Infection

The characteristics of the study population are summarized in Table S1. The majority of women were aged between 26 and 35 years, with a normal BMI. Only a very small proportion of women reported smoking or consuming alcohol. Most participants exhibited normal levels of stress, anxiety, and depression. Prior to the COVID‐19 pandemic, at least 89.3% of the participants received the COVID‐19 vaccine, primarily three doses of CoronaVac (Sinovac), followed by Sinopharm (Beijing). Among the participants, 91.7% experienced a mild COVID‐19 infection that lasted no more than 2 weeks. Additionally, 13.4% (34/253) had one or more general medical conditions and reported a greater incidence of menstrual changes compared to those without such conditions (*p* < 0.001). A total of 44.3% (112/253) reported a history of one or more benign gynecological diseases, while 26.9% (68/253) reported a history of gynecological surgery. The most prevalent symptoms included fever (226, 89.3%), sore throat (185, 73.1%), fatigue (164, 64.8%), cough (157, 62.1%), and headache (141, 55.7%). Of the participants, 54.5% (138/253) received the antipyretic ibuprofen, while 43.5% (110/253) received the “Lianhua Qingwen” capsule. Participants reporting changes in menstruation were more likely to belong to the 36–45 age group, be fertile, and experience a higher incidence of COVID‐19 symptoms along with elevated levels of stress, anxiety, and depression. Women who did not report any menstrual changes were more likely to have good general health and no history of smoking or alcohol consumption.

### 2.3 Statistics of Menstrual Changes in Women With COVID‐19 Infection

Any menstrual change (AnyC) was reported by 155 menstruating women, accounting for 61.3% (155/253) of the primary study population (Table 1). Among these participants, 117 had a regular menstrual cycle of 21–35 days prior to COVID‐19 infection. Changes in menstrual cycle length were the most commonly reported alterations (115/155). 53.5% of participants (83/155) reported longer menstrual cycles while 20.6% (32/155) reported shorter menstrual cycles. Other commonly reported changes included changes in bleeding volume, with 18.1% of participants (28/155) experiencing heavier bleeding and 35.5% (55/155) reporting lighter bleeding. Additionally, 25.8% of participants (40/155) reported prolonged bleeding time, while 14.2% (22/155) reported shortened bleeding duration. Three individuals experienced new onset of dysmenorrhea, whereas 17 experienced worsening dysmenorrhea. Preinfection intermenstrual bleeding was reported by 20.2% (51/253) of participants, while postinfection intermenstrual bleeding was reported by 22.5% (57/253). Among participants who did not report intermenstrual bleeding preinfection, 16.3% (*n* = 33/202) reported intermenstrual bleeding postinfection.

**TABLE 1:** Menstrual changes following COVID‐19 infection.

Any menstrual changes	Questionnaire	Metabolomics
Total	155	51
Menstrual cycle		
Longer menstrual cycle	83 (53.5%)	27 (52.9%)
Shorter menstrual cycle	32 (20.6%)	11 (21.6%)
No change	40 (25.8%)	13 (25.5%)
Duration of menstrual bleeding		
Prolonged bleeding	40 (25.8%)	11 (21.6%)
Shortened bleeding	22 (14.2%)	11 (21.6%)
No change	93 (60.0%)	29 (56.9%)
Menstrual bleeding volume		
Heavier menstrual bleeding	28 (18.1%)	10 (19.6%)
Lighter menstrual bleeding	55 (35.5%)	21 (41.2%)
No change	72 (46.5%)	20 (39.2%)
Degree of dysmenorrhea		
Worsen (including new onset)	20 (12.9%)	8 (15.7%)
Relieve	8 (5.2%)	1 (2.0%)
No change	127 (81.9%)	42 (82.4%)

### 2.4 Clinical Risk Factors Identified for Any Menstrual Changes

We assessed potential risk factors for self‐reported menstrual changes following COVID‐19 infection. As shown in Table 2, we first conducted univariate logistic regression analysis, which revealed potential risk factors such as age (*p* = 0.021 for 26–35 age group; *p* = 0.017 for 36–45 age group), general medical conditions (*p* = 0.002), perceived stress (*p* = 0.020), anxiety (*p* = 0.036), and depression scores (*p* = 0.042), and COVID‐19 symptoms of fatigue and headache, which were significantly associated with menstrual changes (*p* = 0.043 and *p* = 0.049). Analysis of the available data on sex hormones indicated that PRL and AND were significant factors contributing to menstrual changes (*p* = 0.024, and *p* = 0.007). Multivariate logistic regression was conducted using backward stepwise selection to account for various relevant potential confounders; only general medical conditions remained significantly correlated with any menstrual changes (*p* = 0.005).

**TABLE 2:** Unadjusted and adjusted odds of reporting menstrual changes after COVID‐19 infection.

Potential risk factor	Odds of reporting any menstrual changes^a^ after infection (95% CI)	*p*	Adjusted odds of reporting menstrual changes after infection (95% CI)^e^	*p*
Age (years)^b^	1.040 (1.000–1.083)	0.051	/	0.992
Age (years)				
18–25	1.000 (Referent)			
26–35	0.399 (0.183, 0.870)	*0.021*		
36–45	0.418 (0.205, 0.855)	*0.017*		
BMI (kg/m^2^)^b^	0.990 (0.952, 1.030)	0.616	/	0.240
BMI (kg/m^2^)				
Underweight (< 18.5)	1.000 (Referent)			
Normal weight (18.5–23.9)	0.526 (0.196, 1.411)	0.202		
Overweight (24–27.9)	0.439 (0.150, 1.290)	0.135		
Obese (≥ 28)	0.481 (0.160, 1.450)	0.194		
Age of menarche^b^	1.025 (0.842, 1.248)	0.809	/	0.833
Gravidity^b^	1.257 (0.950, 1.663)	0.109	/	0.226
Parity^b^	1.097 (0.738, 1.630)	0.648	/	0.445
Smoking			/	0.405
Never	1.000 (Referent)			
Ever	2.570 (0.283, 23.332)	0.402		
Alcohol intake			/	0.576
Never	1.000 (Referent)			
Past	1.205 (0.491, 2.959)	0.684		
General medical conditions^c^			6.014 (1.738, 20.818)	*0.005*
None	1.000 (Referent)			
1 or more	5.640 (1.921, 16.559)	*0.002*		
Gynecological diseases^d^			/	0.860
None	1.000 (Referent)			
1 or more	1.347 (0.806, 2.249)	0.255		
Gynecological surgery				
None	1.000 (Referent)			
1 or more	1.458 (0.811, 2.621)	0.208		
Vaccine status			**/**	0.122
Not injected	1.000 (Referent)			
Injected	0.220 (0.027, 1.818)	0.160		
Vaccine type				
CoronaVac Sinovac	1.000 (Referent)			
Sinopharm Beijing	1.082 (0.614, 1.907)	0.786		
Others	1.362 (0.240, 7.741)	0.727		
Mixed	1.192 (0.330, 4.301)	0.788		
Vaccine dose				
One	1.000 (Referent)			
Two	1.462 (0.254, 8.401)	0.671		
Three	1.647 (0.323, 8.393)	0.548		
Four	0.600 (0.070, 5.136)	0.641		
Perceived stress score^b^	1.094 (1.014, 1.180)	*0.020*	*/*	0.767
Stress				
Normal (0–14)	1.000 (Referent)			
Mild (15–18)	1.255 (0.112, 14.041)	0.854		
Moderate (19–25)	/	1.000		
Perceived anxiety score^b^	1.098 (1.006, 1.199)	*0.036*	*/*	0.075
Anxiety				
Normal (0–7)	1.000 (Referent)			
Mild (8–9)	6.258 (0.779, 50.272)	0.085		
Moderate (10–14)	3.129 (0.660, 14.829)	0.151		
Severe (15–19)	/	0.999		
Perceived depression score^b^	1.088 (1.003,1.180)	*0.042*	*/*	0.900
Depression				
Normal (0–9)	1.000 (Referent)			
Mild (10–13)	3.296 (0.706, 15.400)	0.129		
Moderate (14–20)	1.978 (0.203, 19.316)	0.558		
COVID‐19 infection				
Asymptomatic infection	1.000 (Referent)			
Mild infection	0.402 (0.044, 3.652)	0.402		
Moderate infection	0.250 (0.023, 2.757)	0.250		
Duration of COVID‐19 infection				
≤ 1 week	1.000 (Referent)			
1–2 weeks	1.174 (0.678, 2.033)	0.566	/	0.844
2–3 weeks	2.231 (0.684, 7.275)	0.183	/	0.196
3–4 weeks	0.687 (0.163, 2.886)	0.608	/	0.620
≥ 4 weeks	0.687 (0.208, 2.262)	0.536	/	0.566
Number of COVID‐19 symptoms	1.056 (0.992, 1.125)	0.090	/	0.649
Most common symptoms				
Fever				
No	1.000 (Referent)			
Yes	1.821 (0.817, 4.058)	0.143		
Sore throat				
No	1.000 (Referent)			
Yes	0.750 (0.419, 1.341)	0.332		
Fatigue			/	0.469
No	1.000 (Referent)	*0.043*		
Yes	1.723 (1.018, 2.918)			
Cough				
No	1.000 (Referent)	0.169		
Yes	0.689 (0.406, 1.171)			
Headache			/	0.087
No	1.000 (Referent)	*0.049*		
Yes	1.672 (1.003, 2.787)			
Medication				
Ibuprofen				
No	1.000 (Referent)			
Yes	0.964 (0.580, 1.602)	0.888		
Acetaminophen				
No	1.000 (Referent)			
Yes	0.686 (0.388, 1.211)	0.194		
Lianhua Qingwen capsule				
No	1.000 (Referent)			
Yes	0.974 (0.585, 1.622)	0.919		
Traditional medicine				
No	1.000 (Referent)			
Yes	1.496 (0.592, 3.780)	0.394		
Sex hormones (not all participants)				
PRL (*n* = 125)	0.958 (0.923, 0.995)	*0.024*		
FSH (*n* = 129)	0.998 (0.971, 1.027)	0.915		
LH (*n* = 129)	1.016 (0.959, 1.076)	0.588		
P (*n* = 78)	1.155 (0.770, 1.731)	0.486		
T (*n* = 112)	0.850 (0.399, 1.807)	0.672		
AND (*n* = 108)	0.901 (0.836, 0.972)	*0.007*		
E_2_ (*n* = 127)	1.003 (0.999, 1.006)	0.148		

Significance of italic *p*‐values < 0.05.

^a^ Any menstrual change means the changes at least one in menstrual cycle length, duration of bleeding, bleeding volume, and dysmenorrhea.

^b^ Continuous variables.

^c^ General medical conditions include cancer, diabetes, hypertension, liver and kidney diseases, thyroid disease, gastrointestinal diseases, gastrointestinal diseases, hyperprolactinemia.

^d^ Gynecological diseases include ovarian cysts, endometrial polyps, uterine myoma, adenomyosis, PCOS, POI, gynecological cancers.

^e^ Adjusted for age, BMI, age of menarche, gravity, parity, smoking, alcohol intake, general medical conditions, gynecological diseases, vaccine status, stress scores (based on DASS‐21), anxiety scores (based on DS‐21), depression scores (based on DS‐21), number of COVID‐19 symptoms, fatigue, headache, and duration of COVID‐19 infection.

### 2.5 Distinct Metabolomic Features Were Observed Among Patients With Different Menstrual Changes

**FIGURE 2:**
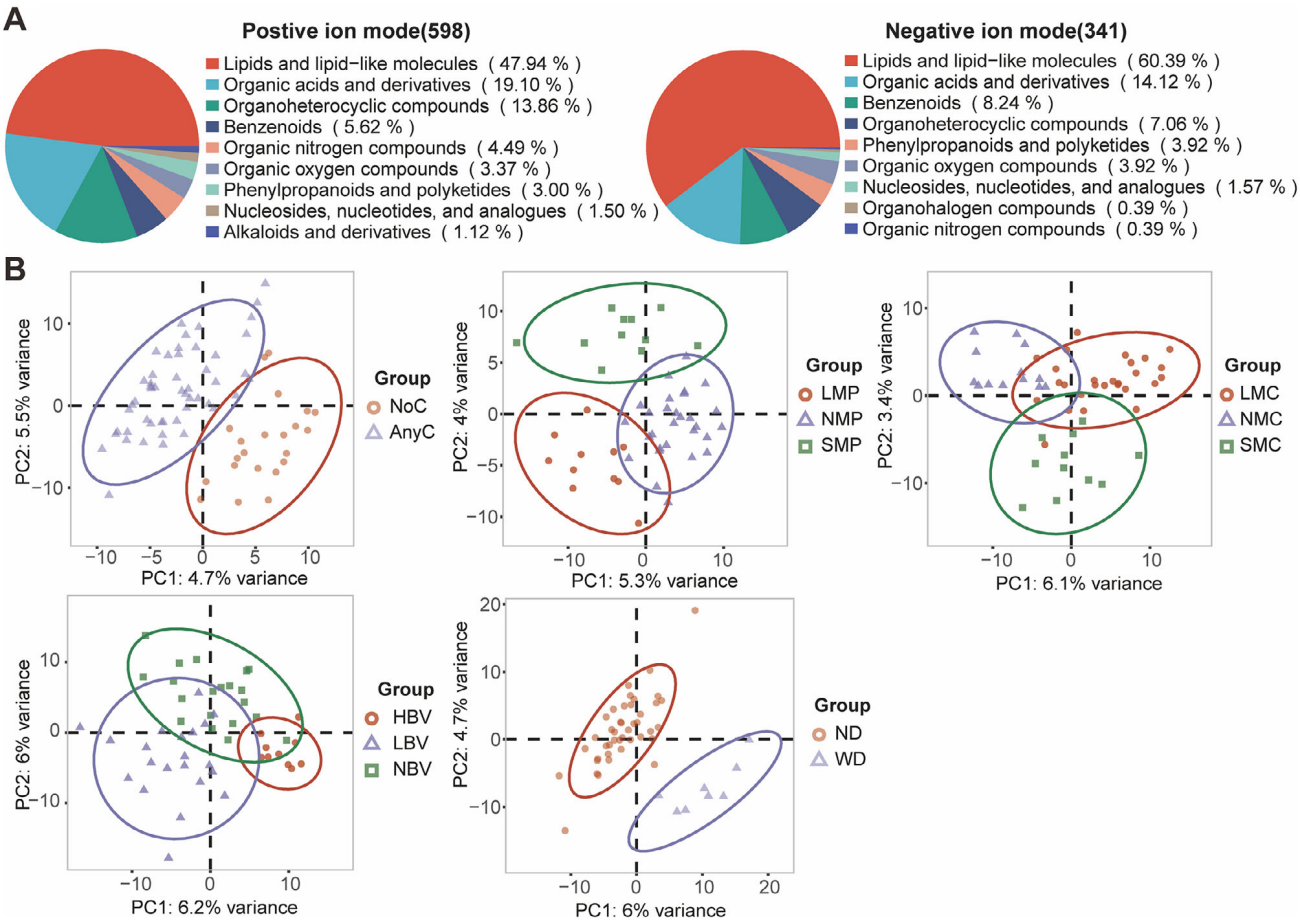
The global metabolite profile among different subgroups. (A) Categories of metabolites detected in either positive (left) or negative (right) ion modes. (B) PLS‐DA of metabolomics data from different menstrual subgroups; PC1 refers to Component 1: the set of coefficients that produces component scores that have the greatest difference between group factors. PC2 refers to Component 2: the second set of coefficients that explains the next highest variability between group factors. Circles represent 95% confidence intervals (CIs). Each point represents individual sample, with points in the same group colored identically. AnyC, any menstrual changes; HBV, heavier bleeding volume; LBV, lighter bleeding volume; LMC, longer menstrual cycle; LMP, longer menstrual period; NA, not available; NBV, nonchanged bleeding volume; ND, nonchanged dysmenorrhea; NMC, nonchanged menstrual cycle; NMP, nonchanged menstrual period; NoC, no menstrual change; SMC, shorter menstrual cycle; SMP, shorter menstrual period; WD, worsen dysmenorrhea.

To investigate the underlying mechanism related to changed menstruation induced by COVID‐19 infection, a cohort of 73 participants was enrolled to characterize the metabolomic patterns. As shown in Table 1 and Figure S1, 51 participants reported AnyC, whereas 22 reported no change (NoC). Among the 51 participants with altered menstrual status, 27 reported a longer menstrual cycle, 11 reported a shorter menstrual period, 21 reported lighter bleeding volume, and only eight reported worsening dysmenorrhea. Based on the menstrual changes, we categorized these participants into five groups. The high Pearson correlation coefficient among quality control (QC) samples indicated the stability and reliability of the detected metabolomic data (Figure S2). In total, 598 metabolites in positive ion mode and 341 metabolites in negative ion mode were identified in this study (Figure 2A). Lipids and lipid‐like molecules were the most abundant compounds in both positive and negative ion mode (Figure 2A). Partial least squares discriminant analysis (PLS‐DA) revealed significant distinctions among the different subgroups (Figure 2B).

**FIGURE 3:**
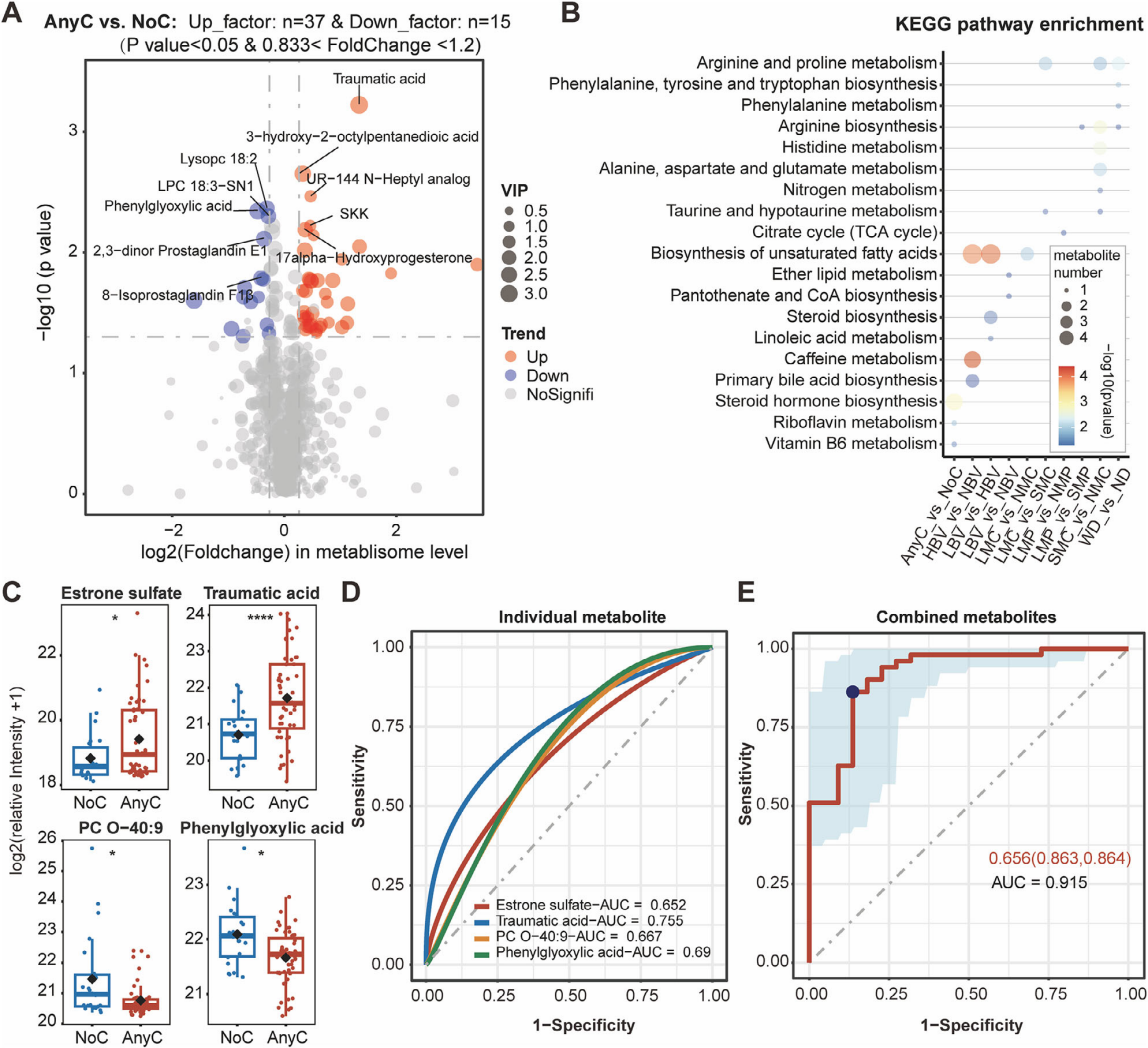
The intergroup significant differential metabolites and corresponding KEGG enrichment pathways for different subgroup comparisons. (A) Volcano plot displaying significant changes in the metabolome between the NoC and AnyC groups. The metabolite names of the top five significantly up‐/downregulated metabolites are shown. The blue points represent upregulated metabolites, the red points represent downregulated metabolites while the gray points indicate insignificant metabolites. The point size is positively correlated with VIP score. (B) Bubble chart displaying KEGG pathways enriched in significant differential metabolites identified in 10 different subgroup comparisons. The *p* value was determined by a hypergeometric test. (C) Box plot illustrating the metabolite levels between the NoC and AnyC groups for four significant differential metabolites applied for constructing an optimized prediction model for menstrual change. Mann–Whitney *U*‐tests were performed for nonparametric comparisons between the two groups. Data are presented as the median and interquartile range (25%–75%). (D) Four ROC curves of the models constructed based on each significantly differential metabolite presented in (C) for the prediction of menstruation with any change. (E) ROC curve of model constructed based on the combination of significant differential metabolites for the prediction of menstruation with any change. The AUC indicates the predictive effectiveness of the combination of four significant differential metabolites. The dot on the broken line in (E) represents the best cut‐off value, and the values in the figure represent the best cutoff (sensitivity, specificity values). The shaded area around the linear regression trend line indicates the 95% CIs. AnyC, any menstrual changes; HBV, heavier bleeding volume; LBV, lighter bleeding volume; LMC, longer menstrual cycle; LMP, longer menstrual period; NA, not available; NBV, nonchanged bleeding volume; ND, nonchanged dysmenorrhea; NMC, nonchanged menstrual cycle; NMP, nonchanged menstrual period; NoC, no menstrual change; SMC, shorter menstrual cycle; SMP, shorter menstrual period; WD, worsen dysmenorrhea.

For subgroup comparison, we identified significant differential metabolites and performed the Kyoto Encyclopedia of Genes and Genomes (KEGG) enrichment analysis (Figure 3A,B; Tables S2 and S3). Notably, the pathways associated with arginine biosynthesis or arginine and proline metabolism were commonly observed in different intergroup comparisons of menstrual status (LMC vs. SMC, SMC vs. NMC, WD vs. ND, LMP vs. SMP; Figure 3B). Meanwhile, pathways associated with the biosynthesis of unsaturated fatty acids were commonly enriched in HBV versus NBV, LBV versus HBV, and LMC versus NMC (Figure 3B). Specifically, a total of 52 differential metabolites were identified between the groups with changed and unchanged menstruation (AnyC vs. NoC). Among these, 37 metabolites, including 3‐phenoxybenzoic acid, traumatic acid, and 18‐β‐glycyrrhetinic acid, were upregulated, and 15 metabolites, such as PC O‐40:9, 10‐nitrolinoleate, and Sakuranin, were downregulated in the AnyC group compared to the NoC group (Figures 3A and S3; Table S2). We observed enrichment of steroid hormone biosynthesis, riboflavin biosynthesis, and vitamin B6 metabolism among differential metabolites in AnyC versus NoC (Figure 3B). In fact, the significant differential metabolites in eight intergroup comparison groups (AnyC vs. NoC, HBV vs. NBV, LBV vs. HBV, LBV vs. NBV, LMC vs. NMC, LMC vs. SMC, LMP vs. NMP, SMC vs. NMC) were commonly involved in the pathway associated with steroid biosynthesis. However, not all of these metabolites reached the significance threshold (*p* < 0.05; Table S3). We further constructed logistic regression models to evaluate the ability to distinguish between individuals with changed and unchanged menstruation based on these differential metabolites. The model, which included phenylglyoxylic acid, PC O‐40, traumatic acid, and estrone sulfate, presented high predictive accuracy in the prediction of AnyC and NoC samples (area under the curve [AUC]: 0.915; Figure 3C–E and Table 3).

**TABLE 3:** Predictive performance indices of significantly changed metabolites in the prediction of samples with any change in menstruation.

Metabolites	Best cutoff	Specificity	Sensitivity	Youden_index	+Likelihood ratio	−Likelihood ratio	Accuracy	AUC (95% CI)	*p* value	90% cutoff	Specificity	Sensitivity	Youden_index	+Likelihood ratio	−Likelihood ratio	Accuracy
PC O‐40:9	0.719	0.545	0.843	0.389	1.85	0.288	0.753	0.667(0.52–0.81)	0.0238	0.787	0.909	0.255	0.164	2.8	0.82	0.452
Estrone sulfate	0.8	0.909	0.373	0.282	4.1	0.69	0.534	0.652(0.52–0.79)	0.0281	0.8	0.909	0.373	0.282	4.1	0.69	0.534
Traumatic acid	0.738	0.864	0.588	0.452	4.31	0.477	0.671	0.755(0.64–0.87)	6.51E−06	0.802	0.909	0.471	0.38	5.18	0.582	0.603
Phenylglyoxylic acid	0.678	0.682	0.725	0.407	2.28	0.403	0.712	0.69(0.55–0.83)	0.00755	0.845	0.909	0.196	0.105	2.16	0.884	0.411
Model	0.656	0.864	0.863	0.726	6.33	0.159	0.863	0.915(0.84–0.99)	6.52E−29	0.87	0.909	0.627	0.537	6.9	0.41	0.712

Model‐four_factors: LOGIT (P (any change in menstruation)) = 47.61 + 0.89 × “estrone sulfate” + 1.22 × “traumatic acid” − 1.53 × “PC O–40:9” − 2.62 × “phenylglyoxylic acid.” Abbreviations: AUC, area under the curve; CI, confidence interval; Inf, infinity. The index in each model were obtained after selecting the cutoff based on the Youden index.

### 2.6 Interaction Observed Among Sex Hormones, Metabolites, and Recovery Duration

**FIGURE 4:**
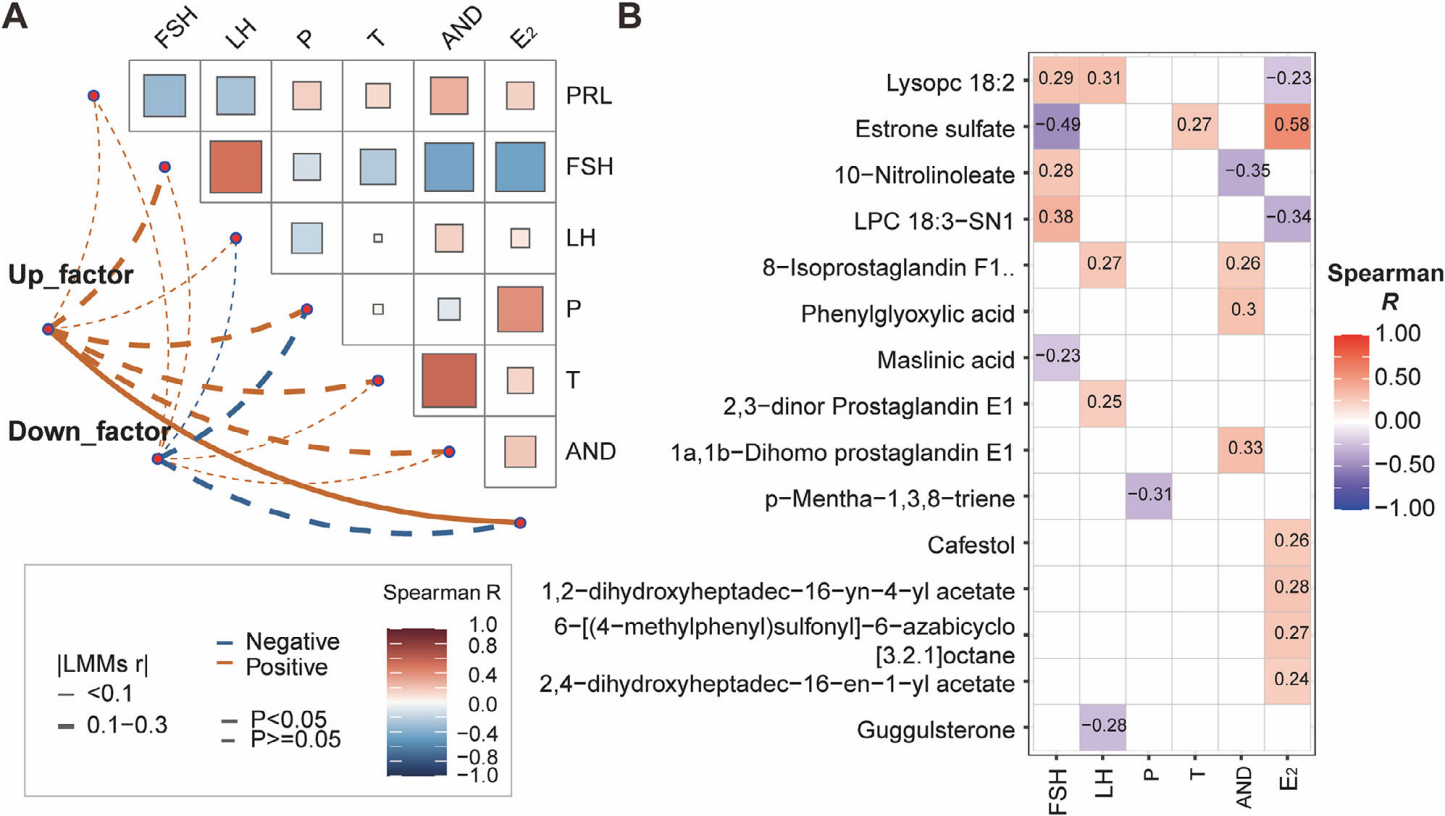
Correlation between sex hormones and differential metabolites profiles between the AnyC and NoC groups. (A) Mantel test of differential metabolite profiles and sex hormones. Mantel's *r* and *p* values are indicated by the color, type, and width of the connecting lines specified in the figure legend. A heatmap on the right‐top displaying the Spearman‐based correlation coefficients (*R*). The color gradient from blue to dark red corresponds positively to the absolute *R* values. (B) Heatmap illustrating the Spearman‐based correlations between metabolite levels and sex hormones. Each row represents metabolite and each column represents a sex hormone. The color gradient from blue to dark red corresponds to the *R* value. Only *R* values with *p* values less than 0.05 are plotted; the others are regarded as null values and excluded or shown as blank here.

**FIGURE 5:**
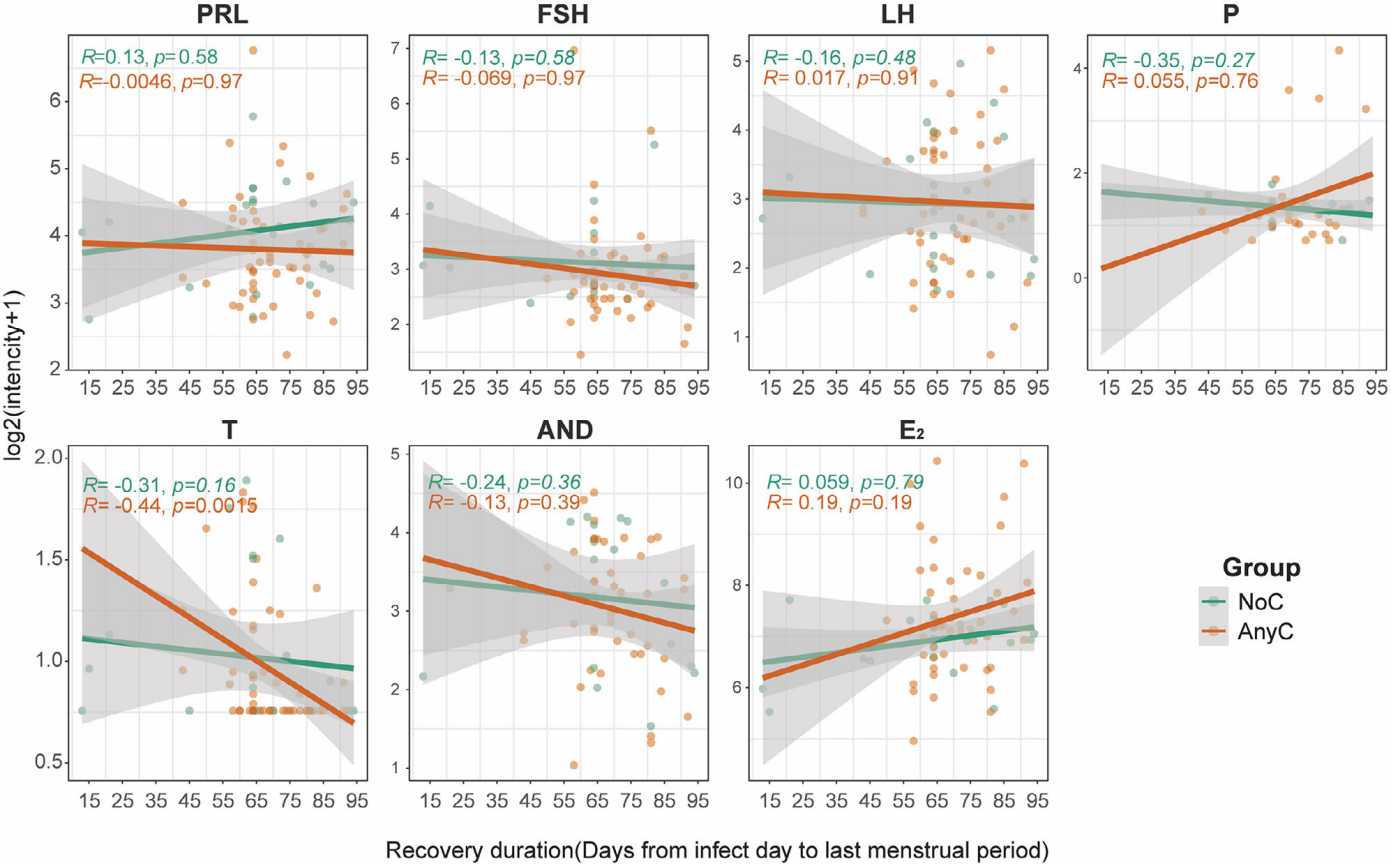
Linear Changes in sex hormone with recovery days. Scatter diagrams depict the levels of sex hormones with recovery days after COVID‐19 infection in the AnyC group and NoC groups. The green/orange straight lines refer to the fitted linear regression lines, with the shaded area referring to the 95% CIs. The correlation coefficients (*R*) and *p* value were calculated using the Spearman correlation test.

To investigate the relationship between the levels of differential metabolites and specific hormone levels, we performed the correlation analysis and found that upregulated metabolites were significantly correlated with the E_2_ level (Mantel test: *R* = 0.242, *p* = 0.015; Figure 4A). The Spearman correlation analysis further revealed a positive correlation between the E_2_ level and five upregulated metabolites (estrone sulfate, cafestol, 1,2‐dihydroxyheptadec‐16‐yn‐4‐yl acetate, 6‐[(4‐methylphenyl) sulfonyl]‐6‐azabicyclo[3.2.1]octane, 2,4‐dihydroxyheptadec‐16‐en‐1‐yl acetate; Figure 4B). Meanwhile, estrone sulfate was also found to be positively correlated with T, but negatively correlated with FSH (Figure 4B). Interestingly, the correlation between recovery duration and changes in sex hormones further revealed that T exhibited downward trend in the AnyC group but remained stable in the NoC group (*p* < 0.05; Figure 5).

## 3 Discussion

COVID‐19 has brought many challenges to the world. In the present study, we examined the effects of COVID‐19 infection on the menstrual health of reproductive‐aged women and compared the metabolic profiles among COVID‐19 participants with different menstrual conditions. Our results found that 61.3% of participants reported at least one kind of menstrual change. Most changes were manifested in prolonged menstrual cycles (83/155), decreased menstrual volume (55/155), and prolonged bleeding time (40/155). Women who reported general medical conditions often experienced more menstrual changes following COVID‐19 infection. The same was observed among women aged 36–45 years old, and those reporting more COVID‐19 symptoms. Phelan et al. reported that 46% of patients experienced changes in their menstrual cycles following the COVID‐19 pandemic [47]. A systematic review performed by Lebar et al. summarized that decreased menstrual volume and a prolonged cycle were mainly reported as consequences of COVID‐19 infection [22], consistent with our findings. Li et al. also reported that 20% of patients with COVID‐19 exhibited decreased menstrual volume and 18% of patients experienced prolonged cycles, with severely ill patients experiencing even longer menstrual cycles [27]. However, most participants in our study had mild infections, thus, we are lacking data on severe COVID‐19 infection. Khan et al. reported that changes in menstrual cycles were positively correlated with an increased number of COVID‐19 symptoms in infected participants [25]. Our study corroborated this finding; however, the comparison was not statistically significant (8.08 and 7.15, *p* = 0.088). When comparing COVID‐19 symptoms, individuals reporting any menstrual changes were more likely to report fatigue, headache, and decreased or loss of interest (*p* < 0.05), compared to participants who did not report menstrual changes.

Numerous studies have reported menstrual changes following COVID‐19 vaccination [48, 49, 50, 51]. Jensen et al. reported that 30% of menstruating women experienced menstrual changes following COVID‐19 vaccination [52]. A study by Wang et al. found that women vaccinated against COVID‐19 may experience a short‐term increase in cycle length compared to their unvaccinated counterparts [53]. Individuals with regular menstrual cycles reported changes in their menstrual periods and flow after receiving the vaccine [54]. Moreover, menstruating females who received the COVID‐19 vaccination reported experiencing worse menstrual pain, heavier flow, and prolonged bleeding [55]. A study by Wong et al. did not establish a relationship between menstrual irregularities together with vaginal bleeding and the COVID‐19 vaccination [56]. As of July 23, 2022, the coverage rates for the first dose of the COVID‐19 vaccine, complete vaccination, and second dose were 92.1%, 89.7%, and 71.7%, respectively. In the present study, at least 89.3% of participants had already received the COVID‐19 vaccine. We found no association between menstrual changes and vaccine status.

Importantly, many studies have described COVID‐19‐related stress and anxiety, which may explain the association between COVID‐19 infection and menstrual irregularities [57, 58]. Payne et al. reported that COVID‐19‐related stress and distress were positively associated with increased dysmenorrhea and more menstrual symptoms [59]. Aolymat et al. described the significant association between DASS‐21 scores and heavy bleeding as well as amenorrhea [26]. Women with high COVID‐related stress scores were more likely to report a prolonged menstrual duration and heavy bleeding [60]. However, some studies have reported mixed results [61]. COVID‐19‐related anxiety and stress heightened women's menstrual symptoms while decreasing the length and volume of bleeding [62]. A study by Nguyen et al. found that women reporting higher stress levels during the COVID‐19 pandemic were not more likely to experience menstrual changes [21]. Similarly, Cherenack et al. also failed to find associations between perceived stress, depression, loneliness, and menstrual irregularities [63]. We first categorized stress, anxiety, and depression into three degrees based on DASS‐21 scores. Nearly 90% of participants exhibited normal levels of stress, anxiety, and depression. Mental status did not appear to be significantly related to menstrual changes. However, when we analyzed the impact of COVID‐19‐related mental health on menstruation based on their DASS‐21 scores, we found that higher stress, anxiety, and depression scores were associated with increased self‐reported menstrual changes (*p* < 0.05), consistent with the literature.

It is well known that sex hormone levels influence menstruation. In the present study, women with menstrual changes had significantly lower serum PRL and T levels, suggesting abnormal reproductive hormone profiles compared to COVID‐19‐infected participants who did not report menstrual changes. Ding et al. reported that women affected by COVID‐19 had significantly lower serum AMH levels and higher T/PRL levels compared to uninfected healthy women, suggesting that COVID‐19 might have a deleterious effect on ovarian reserve and endocrine function [64]. Additionally, they found significant differences in T levels among several laboratory characteristics of COVID‐19 patients [64]. However, a study by Li et al. reported no statistically significant differences in sex hormone concentrations or ovarian reserve between COVID‐19 patients of childbearing age and controls, as well as in subgroup analysis based on menstrual changes [27]. In addition, we investigated the relationship between the levels of differential metabolites and specific hormone levels, finding that upregulated metabolites were significantly correlated with E_2_ levels. Estrone sulfate, an E_2_ precursor, was positively correlated with E_2_ and T, but negatively correlated with FSH. A previous study demonstrated that the cycle phase is associated with variations in E_2_ formation due to the metabolism of estrone sulfate [65]. Further studies should be conducted to explore the role of estrone sulfate in regulating hormones and menstruation. The difference was that all participants analyzed in our study were COVID‐19‐infected women. Not all infected patients had changed serum T levels, but lower serum T levels may contribute to menstrual change [66].

Cherenack et al. demonstrated that detectable COVID‐19 IgG antibodies were associated with menstrual irregularities regardless of vaccination status [63]. The association between COVID‐19 infection and menstrual changes may also be attributed to alterations in sex hormones and immune responses [64]. In the present study, we performed a metabolic analysis to unravel the underlying mechanisms by which COVID‐19 infection affects menstruation. We identified 37 upregulated metabolites and 15 downregulated metabolites, with steroid hormone biosynthesis being the most significantly altered metabolic pathway. Menstruation is a steroid‐regulated process [11]. These altered metabolites may play a role in sex hormone changes, thus inducing menstrual changes. Further analyses indicated that the upregulated factors were significantly associated with the E_2_ levels, providing evidence for this hypothesis.

### 3.1 Limitation

This study investigated the impact of COVID‐19 infection on women's menstrual health, controlling for key potential confounders such as the COVID‐19 vaccine and mental health. Metabolomics was employed to explore potential causal relationships and to better understand the effects of COVID‐19 on ovarian function. However, despite these strengths, the study still has several limitations. First, this survey was conducted in a general hospital setting, with nearly half of the participants being outpatient who sought care for menstrual changes. This sample may not accurately represent the population prevalence of postinfection menstrual changes, potentially leading to an overestimation of their proportion. Second, since the study aimed to investigate menstrual changes following COVID‐19 infection, noninfected women were excluded. This design limits the ability to explore the causal relationship between COVID‐19 infection and menstrual changes. Third, although potential biases may arise from factors such as vaccine status, type, dose, COVID‐19 infection, and duration of infection, the small sample size in certain subgroups limits our ability to conduct reliable subgroup analyses, and their influence may need further validation from additional studies. Furthermore, not all participants underwent blood tests, and some specimens were collected regardless of menstrual cycle status, which may influence the basal levels of hormones and affect the ability to detect significant changes in FSH and LH. Therefore, larger sample sizes and long‐term prospective cohorts are needed to confirm these findings and enhance our understanding of the impact of COVID‐19 on menstrual health.

## 4 Conclusion

Overall, this study observed menstrual changes following COVID‐19 infection in some participants, primarily manifested as prolonged menstrual cycles and lighter bleeding. Additionally, infection‐related menstrual changes were along with metabolic disorders involving steroid hormone biosynthesis and correlated with specific hormone levels. Our study highlights the adverse impact of the pandemic on women's reproductive well‐being. Further research is warranted to assess the long‐term consequences of COVID‐19 infection on menstrual health and to elucidate the underlying mechanisms.

## 5 Materials and Methods

### 5.1 Study Population

The prospective study was initiated in February 2023 and lasted for 4 months to examine whether COVID‐19 infection was associated with menstrual changes at Peking University Third Hospital. Information was collected using a questionnaire emphasizing the anonymity and confidentiality of the participants. The content of the questionnaire was validated through pilot testing to ensure that the survey was clear and reliable. Two standardized, trained investigators were responsible for the collection and analysis of data to minimize information bias. The researchers distributed 350 questionnaires, and 307 patients were ultimately recruited from the gynecology clinic (the response rate was 87.7%). Since information on menstrual changes was collected only from women after COVID‐19 infection, noninfected women were excluded. The inclusion criteria were as follows: (1) menstruating women aged 18–45 years; (2) confirmed cases or clinically diagnosed cases of COVID‐19 infections (based on the diagnostic criteria outlined in the “Diagnostic and treatment protocol for novel coronavirus infections (Trial 10th edition)”); the exclusion criteria included (1) pregnant, lactating and menopausal women; (2) those suffering from other diseases that may cause irregular menstruation (e.g., ovarian tumors, functional hypothalamic amenorrhea, thyroid disease, hyperprolactinemia, early onset ovarian insufficiency, polycystic ovary syndrome, etc.); (3) those who had taken oral or transdermal estrogen‐containing products such as contraceptives or menopausal hormone treatments; (4) those unable to cooperate with the survey. The study cohort was established immediately following the first rapid infection surge after the termination of “dynamic zero‐COVID policy” on December 7, 2022. Most participants experienced their first infection, effectively eliminating the confounding effects of reinfection and making these data of unique value for assessing the postrecovery impact of COVID‐19.

Among these, 73 participants underwent laboratory tests for routine medical purposes, and their blood samples were utilized for metabolomics analysis. All surveys collected self‐reported information regarding COVID‐19 infection and symptoms, COVID‐19 vaccination, and changes in menstrual cycles, menstrual duration, and menstrual flow. The baseline survey also collected self‐reported information on the Depression‐Anxiety‐Stress Scale (Chinese version) (DASS‐21) [67].

### 5.2 Definition of Measures

COVID‐19 infection was defined as a positive test for a real‐time reverse‐transcriptase–polymerase chain reaction (RT‐PCR) assay, an antigen test, or the presence of typical symptoms. Patients were classified as mild or severe according to the Diagnosis and Treatment Protocol for Novel Coronavirus Pneumonia (Trial Version 10) as follows: (i) Mild: mild clinical symptoms with or without typical CT imaging of viral pneumonia; (ii) Severe: oxygen saturation ≤ 95% at rest, or respiratory distress with respiration rate < 30 breaths per min, or arterial partial oxygen pressure (PaO_2_)/oxygen absorption concentration (FiO_2_) ≤ 300 mm Hg, or respiratory failure requiring mechanical ventilation, or shock, or organ failure necessitating intensive care. Menstrual changes were reported using categorical responses such as “Longer than before,” “Shorter than before,” or “Same as before.” In addition to questions about any menstrual changes, participants were also asked to recall instances of intermenstrual bleeding both before and after the infection.

### 5.3 Untargeted Metabolomics

A total of 500 µL of serum was collected from each woman for subsequent untargeted metabolomic analysis. The samples were mixed with prechilled 80% methanol, placed on ice for 5 min, and subsequently centrifuged. A fraction of the resulting supernatant was diluted with LC–MS grade water, achieving a final methanol concentration of 53%.

The supernatant was then analyzed using a Vanquish UHPLC system (Thermo Fisher, Germany) linked to either an Orbitrap Q Exactive HF mass spectrometer or an Orbitrap Q Exactive HF‐X mass spectrometer (Thermo Fisher, Germany) for UHPLC–MS/MS analysis. Samples were eluted from a Hypersil Gold column (100 × 2.1 mm, 1.9 µm) using a 12‐min linear gradient at a flow rate of 0.2 mL/min. The eluents for the positive polarity mode were eluent A (0.1% formic acid in water) and B (methanol). The eluents for the negative polarity mode were eluent A (5 mM ammonium acetate, pH 9.0) and B (methanol). The gradient program was set as follows: 2% B, 1.5 min; 2%–85% B, 3 min; 85%–100% B, 10 min; 100%–2% B, 10.1 min; 2% B, 12 min. The Q Exactive HF mass spectrometer functioned in both positive and negative polarity modes, applying a spray voltage of 3.5 kV. The capillary temperature was maintained at 320°C, with a sheath gas flow rate set at 35 psi and an auxiliary gas flow rate of 10 L/min. The S‐lens RF level was adjusted to 60, while the auxiliary gas heater was kept at 350°C.

The raw data obtained from UHPLC–MS/MS analysis were analyzed with Compound Discoverer 3.3 (CD3.3, ThermoFisher) for peak alignment, peak picking, and quantitation of each metabolite. Statistical analyses were conducted using the statistical software R (version 4.3.1). The metabolites were annotated with multiple databases, including the KEGG, HMDB, and LIPID Maps.

### 5.4 Statistical Analysis

All statistical analyses were performed using SPSS Statistics (version 25) and the statistical software R (version 4.3.1). Characteristics of the participants were presented with descriptive statistics (counts and frequencies). Continuous variables were checked for normality and expressed as mean ± SD when normally distributed or median (interquartile range, IQR) when not normally distributed. Categorical variables were reported as counts and percentages (%). Student's *t*‐test and Mann–Whitney *U*‐tests (nonparametric) were applied to continuous variables as appropriate, while Pearson's chi‐square tests or Fisher's exact tests were performed for categorical variables. Univariable and multiple logistic regression were conducted to identify the potential risk factors for reporting any menstrual change following COVID‐19 infection. Spearman's‐based correlation test was used to determine the correlation coefficient and *p* value in the correlation analysis between sex hormones and recovery days or metabolites, using the functions “cor.test” and “cor” in R package “stats” (version 4.3.1). PLS‐DAs in profiling the plasma metabolome and calculating the Variable Importance in Projection (VIP) scores were performed via the function “plsda” in the R package “mixOmics” (version 6.26.0) [68]. For the classification between two groups, the metabolites with VIP > 1 and fold change (FC) > 1.2 or FC < 0.833 and *p* value < 0.05 were regarded as significant differential metabolites. For all tests, a *p* value < 0.05 was considered statistically significant. Binomial logistic regression analysis was performed on log2‐transformed relative intensities of significant differential metabolites, either single or multiple, using the “glm” function from the R package “stats” (version 4.3.1). The optimal combination of metabolites with the lowest Akaike information criterion (AIC) value for fitting new regression models was determined using the “stepAIC” function from the R package “MASS” [69] (version 7.3‐60) with backward selection. Model predictions were obtained using the “Predict” function from the R package “car” (version 3.1‐2) [70]. Receiver operating characteristic (ROC) curves were constructed to evaluate the discriminatory capacity of metabolites between NoC and AnyC group using the “roc” and “ggroc” functions from the R package “pROC” (version 1.18.5) [71]. The 95% confidence interval (CI) of the AUC was calculated using the “ci.auc” function with the default “delong” method in the pROC package (version 1.18.5) [71].

## Author Contributions

Dr. Jie Qiao and Dr. Wei Chen have full access to all of the data in the study and take responsibility for the integrity of the data and the accuracy of the data analysis.

*Concept and design*: Jie Qiao, Wei Wang, Yuanyuan Wang, Liying Yan, Rong Li.

*Acquisition, analysis, or interpretation of data*: Wei Wang, Manfei Si, Wei Chen, Xinyu Qi, Hongxia Hu, Xiaole Sun, Juyan Liang, Jianghua Zhou, Xianmin Bi, Wei Zhao.

*Drafting of the manuscript*: Manfei Si, Wei Chen.

*Critical review of the manuscript for important intellectual content*: Wei Wang, Manfei Si, Xinyu Qi, Hongxia Hu, Xiaole Sun, Juyan Liang, Jianghua Zhou, Xianmin Bi, Wei Zhao, Yuanyuan Wang, Liying Yan, Rong Li, Wei Chen, Jie Qiao.

*Statistical analysis*: Wei Chen, Manfei Si.

*Obtained funding*: Wei Chen, Jie Qiao, Manfei Si.

*Supervision*: Jie Qiao, Wei Chen.

All the authors read and approved the final manuscript.

## Ethics Statement

Written informed consents were obtained from participants for research purposes prior to the study, in accordance with the Declaration of Helsinki. The protocol for this study was reviewed and approved by the Ethics Committee of Peking University Third Hospital (Approval No. M2023081).

## Consent

The authors have nothing to report.

## Conflicts of Interest

The authors declare no conflicts of interest.

## Supporting information



Supporting Information

Supporting Information

## Data Availability

The raw metabolism data in this study have been deposited in the OMIX database of the National Genomics Data Center (https://ngdc.cncb.ac.cn/omix) under accession number OMIX008741, and is available from the corresponding author upon request.
