# Supplementary material for: The Clinical and Metabolic Profiles in Menstrual Changes Among Reproductive‐Aged Women Post‐COVID‐19
Source: MedComm (2020). 2025 Jun 11;6(6):e70240. doi: 10.1002/mco2.70240 (PMC12152425; doi:10.1002/mco2.70240)
Supplement: Supplementary file 1 — Supporting Information [file MCO2-6-e70240-s001.docx]

**The Clinical and Metabolic Profiles in Menstrual Changes among Reproductive-Aged Women post-COVID-19**

**Running Head**

Menstrual metabolism disorder after COVID-19

Wei Wang, MD^1,2,†^, Manfei Si, PhD^2,3,4, †^, Xinyu Qi, PhD^2,3,4^, Hongxia Hu, MS^1,2^, Xiaole Sun, MS^1,2^, Juyan Liang, MS^1,2^, Jianghua Zhou, MS^1,2^, Xianmin Bi, MS^1,2^, Wei Zhao, MD^5^, Yuanyuan wang, PhD^2,3,4,^ , Liying Yan, PhD^2,3,4^ , Rong Li, PhD^2,3,4^ , Wei Chen, PhD^1,2,*^, Jie Qiao, PhD^1,2,3,4,6,7,*^

1. Department of Obstetrics and Gynecology, Peking University Third Hospital, Beijing 100191, China;
2. National Clinical Research Center for Obstetrics and Gynecology (Peking University Third Hospital), Beijing 100191, China;
3. Center for Reproductive Medicine, Department of Obstetrics and Gynecology, Peking University Third Hospital, Beijing 100191, China;
4. State Key Laboratory of Female Fertility Promotion，Department of Obstetrics and Gynecology, Peking University Third Hospital;
5. Physical Examination Center, Peking University Third Hospital, Beijing 100191, China;
6. Beijing Advanced Innovation Center for Genomics, Beijing 100191, China;
7. Peking-Tsinghua Center for Life Sciences, Peking University, Beijing 100191, China

* Corresponding Authors: Jie Qiao, PhD, and Wei Chen, PhD, Center for Reproductive Medicine, Department of Obstetrics and Gynecology, Peking University Third Hospital, No. 49 North Huayuan Road, Haidian District, Beijing 100191, China ([jie.qiao@263.net](mailto:jie.qiao@263.net) and [weichen-cls@pku.edu.cn](mailto:weichen-cls@pku.edu.cn) ).

† Wei Wang and Manfei Si are contributed equally.

**Supplementary Figures**


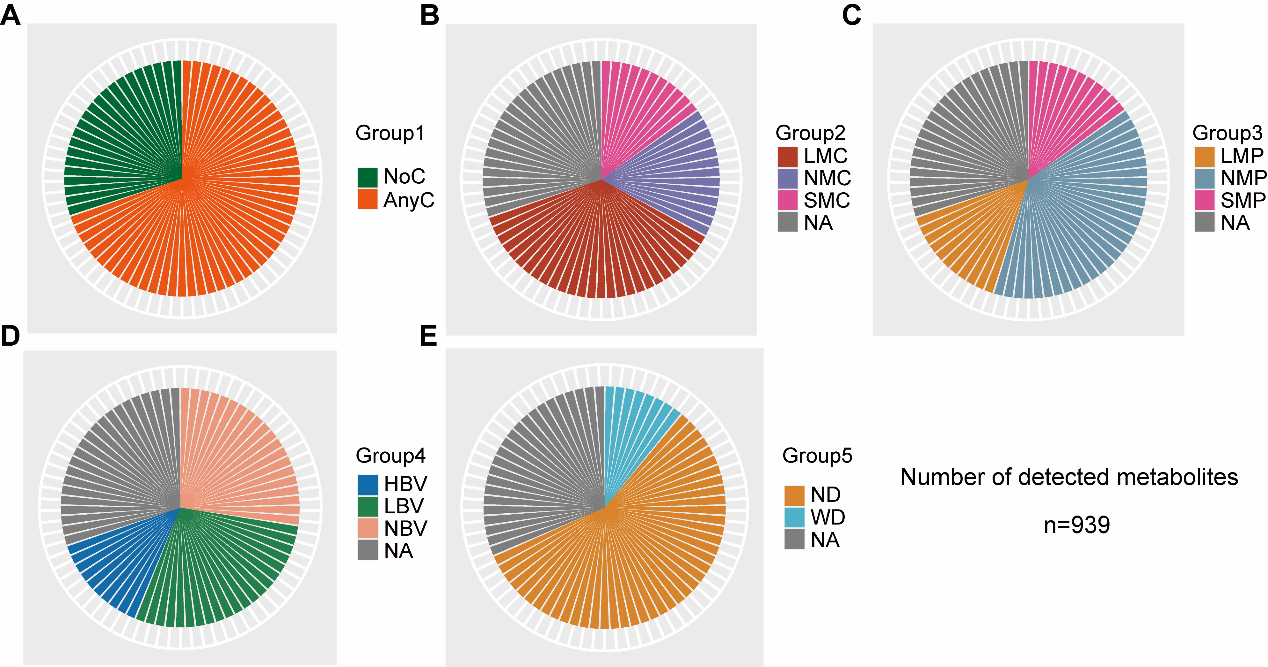


**Figure S1. The number of participants in different subgroups**. **Abbreviations**: NoC, no menstrual change; AnyC, any menstrual changes; LMC, longer menstrual cycle; NMC, non-changed menstrual cycle; SMC, shorter menstrual cycle; NA, not available; LMP, longer menstrual period; NMP, non-changed menstrual period; SMP, shorter menstrual period; HBV, heavier bleeding volume; NBV, non-changed bleeding volume; LBV, lighter bleeding volume; ND, non-changed dysmenorrhea; WD, worsen dysmenorrhea.


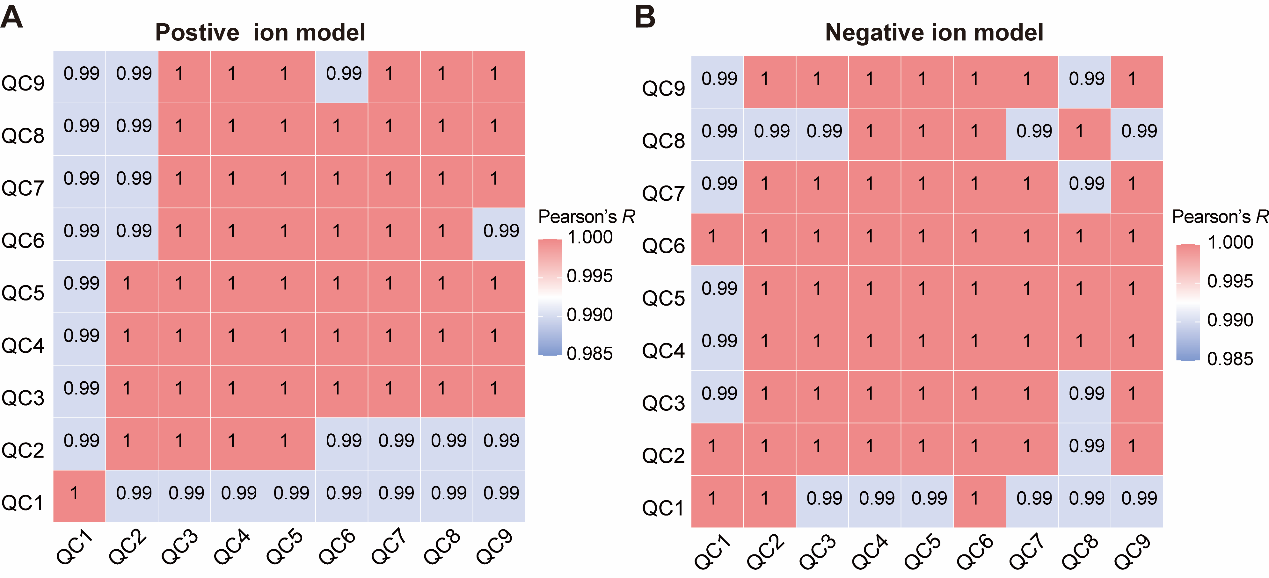


**Figure** S**2**. The Pearson correlation coefficient(*R*) among QC samples (**A:** positive ion model; **B:** negative ion model)


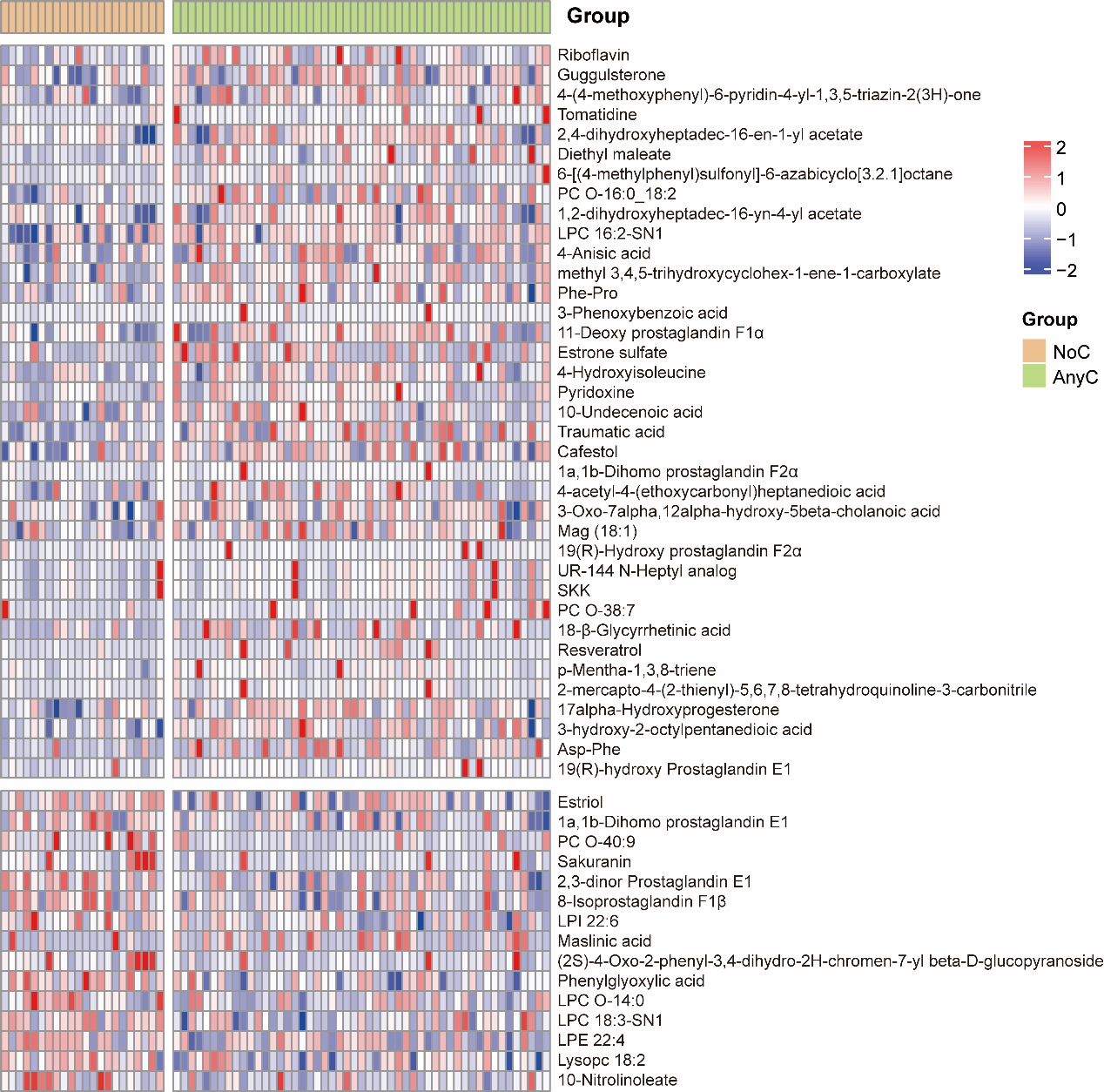


**Figure S3.** Heatmap presenting showing the row-scaled metabolite level between NoC and AnyC groups. The color gradient from blue to dark red correlates positively with the relative level.

**Supplementary tables provided in zip file**

**Table S1.** Characteristics of the study population (menstruating women).

**Table S2:** The significantly differential changed metabolites identified in each comparison group

**Table S3:** The KEGG enrichment analysis results for significantly differential changed metabolites in each comparison group
